# Sarcoma in a Patient with Laryngeal Papillomatosis: A Case Report

**DOI:** 10.22038/IJORL.2021.57188.2967

**Published:** 2022-01

**Authors:** Eleana Tzoi, Konstantinos Garefis, Vasilios Nikolaidis, Ourania Batsi, Konstantinos Markou

**Affiliations:** 1 *Department of Head and Neck Surgery, Aristotle University of Thessaloniki, Papageorgiou Hospital, Thessaloniki, Greece.*; 2 *Department of Pathology, Papageorgiou Hospital, Thessaloniki, Greece*

**Keywords:** HPV, Mitomycin, Recurrent respiratory papillomatosis, Sarcoma

## Abstract

**Introduction:**

Recurrent respiratory papillomatosis (RRP) is classically described as a benign neoplasm of the larynx. Nevertheless, transformation to dysplasia and invasive carcinoma can occur. Sarcoma of the larynx is rare. Here, we present a case of sarcoma in a patient repeatedly treated for RRP.

**Case Report:**

We report a case of a 73- year old Caucasian male diagnosed with adult-onset recurrent respiratory papillomatosis (AORRP) at the age of 63y. o. During the previous 10 years, he underwent multiple surgeries. In the last therapeutic intervention, he was treated with laser excision of the papilloma and topical mitomycin application. Two months after treatment, papilloma recurred and sarcoma was diagnosed.

**Conclusions:**

RRP is a benign lesion.  Affected patients usually require multiple interventions. It rarely degenerates to malignancy. Sarcoma in the larynx in the presence of RRP is a rare case. Extended surgical removal remains the treatment of choice. Adjuvant therapies consist of chemotherapy and radiation and are reserved for unresectable or recurrent cases.

## Introduction

Recurrent respiratory papillomatosis (RRP) is characterized by multiple recurring papillomas throughout the respiratory tract, predominantly in the larynx and the trachea. The risk of malignant transformation is approximately 3–7%. Laryngeal sarcoma is an extremely rare malignancy and accounts for <1% of all malignant laryngeal tumors. We present the first clinical report in the literature of sarcoma on a patient with laryngeal papillomatosis. 

## Case Report

**Fig 1:**
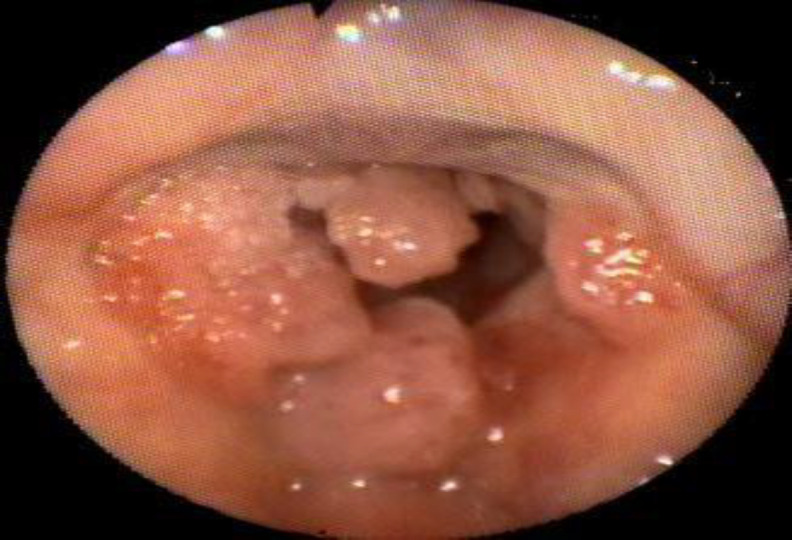
Fiberoptic endoscopic image of papillomatous lesion. Flexible endoscopic examination of the larynx preoperatively showing the papillomatous lesion that occupies both vocal cords, posterior and anterior commissure

**Fig 2:**
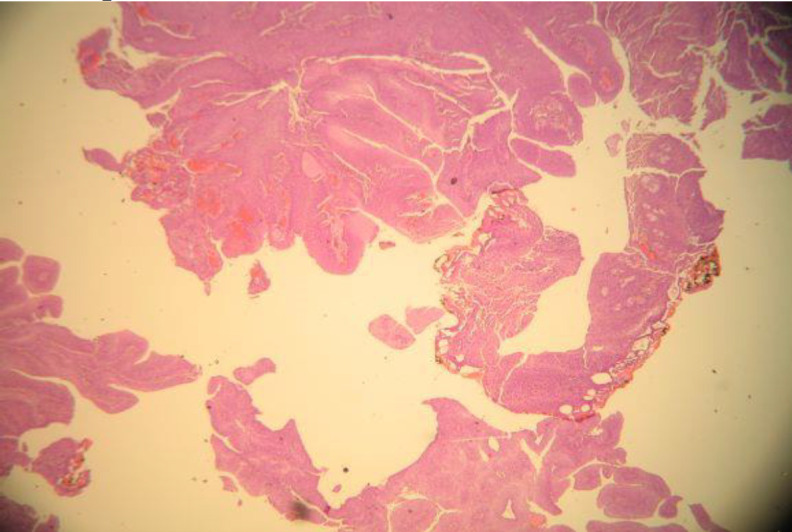
Histopathological examination. Fragments of papilloma are characteristically composed of a squamous epithelium covering fibrovascular cores (white arrows) (H&E, Χ40).

A 73- year old Caucasian male suffering from RRP, initially diagnosed 10 years ago, was subjected to periodical endoscopic examination for his disease. There was no history of smoking and alcohol consumption. In a period of 10 years, he underwent multiple surgeries including laser and cryosurgical removal of the papillomatous lesion.He was also vaccinated with Gardasil HPV vaccine. In his last surgery, he was treated with laser removal of the papillomatous lesion and topically mitomycin application (Figure 1). The histological description was that of papilloma (Figure 2).

**Fig 3 A-C:**
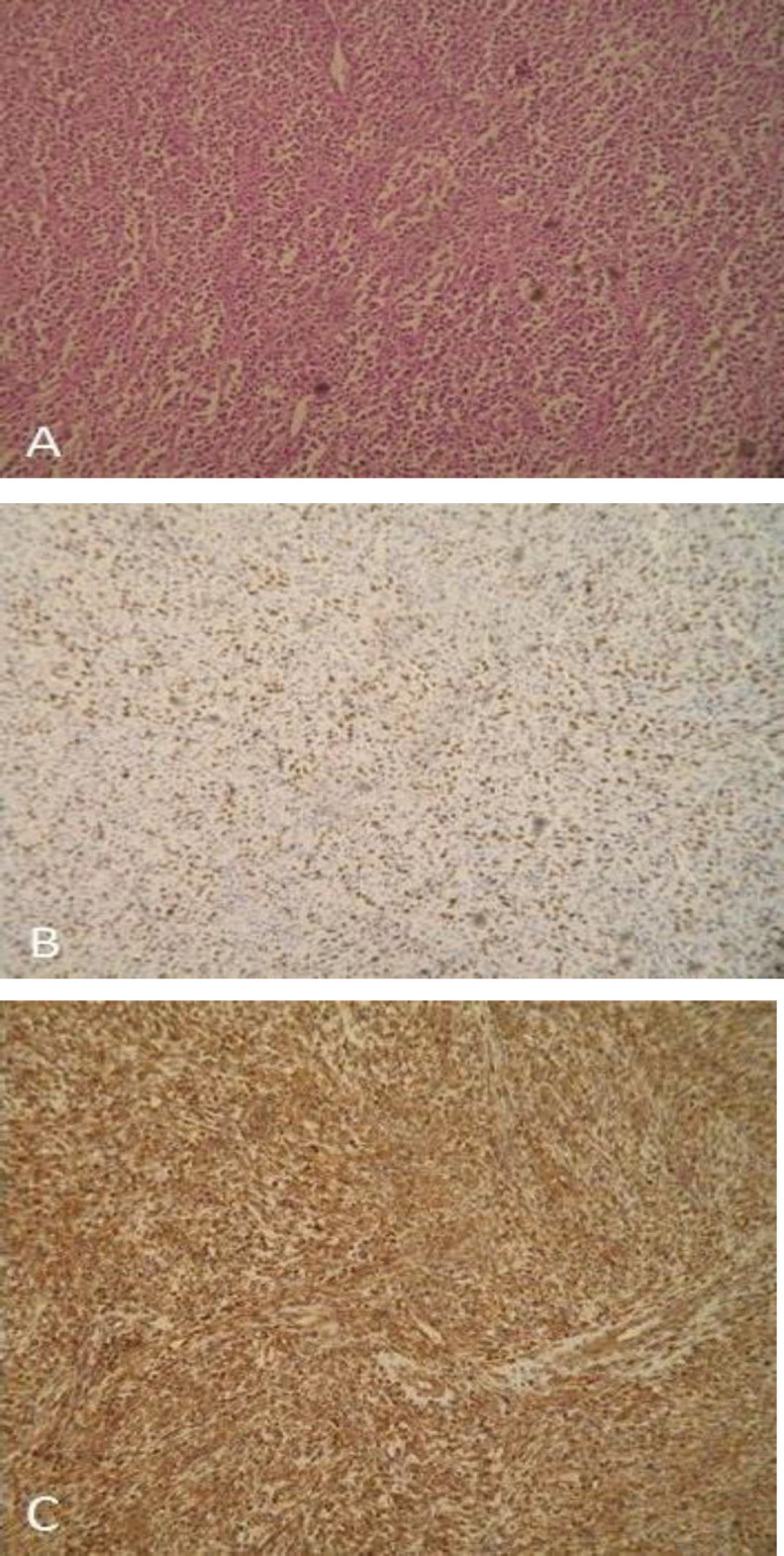
Histopathological and immuno- histochemically examinations. (A)Sarcoma cells are distributed perpendicularly or in interconnected bundles (H&E, Χ10), (B) Ki-67 + about 40%, (C) Vimentin marker +. H&E, hematoxylin and eosin

A month later, the patient turned up at his local Emergency Department with progressive dyspnea. He underwent an urgent tracheotomy. Fiberoptic nasopharyngeal endoscopy in our ENT department showed a large mass occupying the oropharynx and laryngeal vestibule. Microlaryngoscopy and biopsy were carried out under general anesthesia. Histopathological and immunohistochemical examinations showed undifferentiated sarcoma grade 2 (Figure 3 A-C). 

**Fig 4 A-B:**
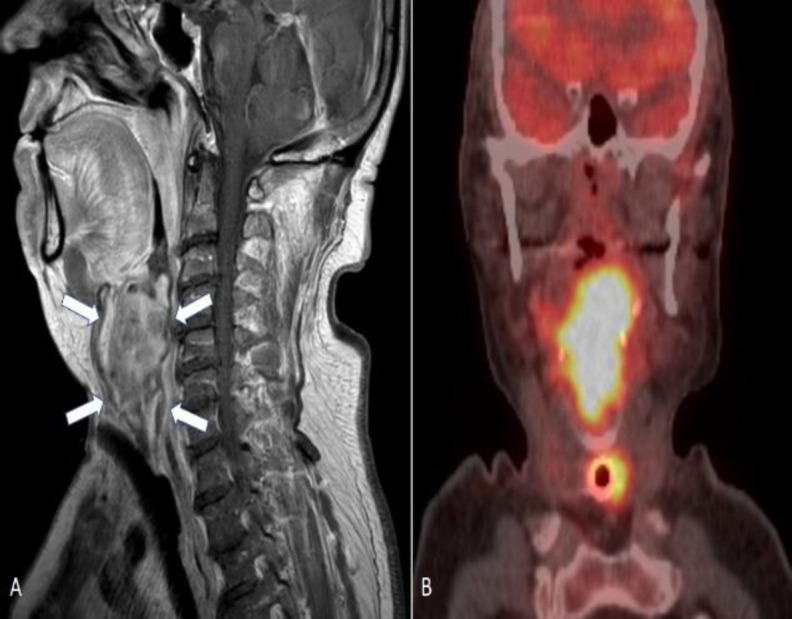
Magnetic resonance (Α) and Combined FDG-positron emission tomography (PET)/ computed tomography (CT) (B) images. (A) Sagittal MRI scan showing the mass (white arrow) that occupies oropharynx, hypopharynx, glottic and subglottic area. (B) Coronal PET/CT imaging - the tumor appears as a hypermetabolic lesion (hot spot) [Time-of-flight (TOF) reconstruction]

Magnetic resonance imaging (MRI) of the neck (Figure 4 A), computed tomography scan (CT) of thorax and abdomen, and a fluorodeoxyglucose (FDG) positron emission tomography (PET)/CT scan were performed (Figure 4 B).

The radiological findings were a large mass, 46.1X35.2X40.2mm in size, arising from the larynx, one right cervical lymph node involvement and no distant metastasis.

**Fig 5 A-B:**
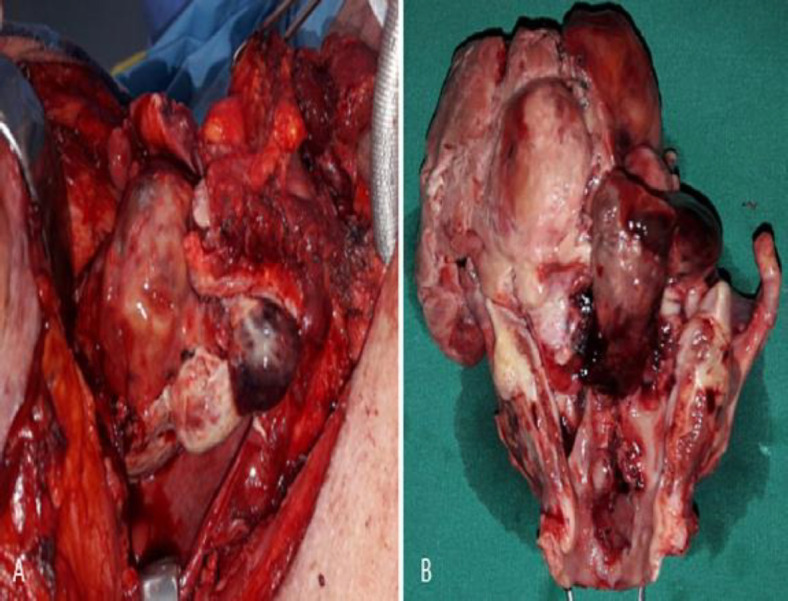
(A) Intraoperative and (B) postoperative image during total laryngectomy showing the extension of the sarcoma

PET/CT scan showed intense FDG uptake of the lesion with SUVmax of 35.6. After evaluation by the multidisciplinary team (MDT), total laryngectomy, total thyroidectomy, and bilateral elective neck dissection (levels II-III-IV-V) were performed (Figure 5 A-B). 

The histopathological and immunohisto- chemical examinations confirmed the former diagnosis showing a pT3N1M0 sarcoma. The excision appeared to be complete. One month later, the patient had a recurrence of the mass in the oropharynx with a new biopsy confirming sarcoma recurrence. He was treated with four cycles of adjuvant chemotherapy (doxorubicin, ifosfamide and mesna) and palliative radiotherapy. The patient died seven months after the total laryngectomy due to locally extensive disease. 

## Discussion

Infection by HPV subtypes 6 and 11 accounts for most cases of RRP (1). Malignant transformation is attributed mainly to types 16 and 18. Squamous cell carcinoma is the most common transformation (2,3). It is currently unknown if HPV vaccination can prevent oral HPV infections that lead to cancer or papillomatosis in the upper airway (1,2).

Mitomycin C, the agent administered to this patient, is widely used as an antibacterial, chemotherapeutic agent, and fibroblast inhibitor (4). It has been used in RRP, resulting at clinical remission after topical application. There is one report in the literature that indicates that topical mitomycin C application may have caused laryngeal squamous cell carcinoma because of its alkylating properties, but there are no current reports that mitomycin application can cause laryngeal sarcoma (5,6). 

Head and neck is an uncommon site for soft tissue sarcoma and accounts for <10% of all sarcomas (7,8). 

Smoking, alcohol, and radiation are well-established predisposing factors in carcinosarcoma/sarcomatoid carcinomas of the larynx. Surgery, with either local excision or wide excision (laryngectomy), remains the gold standard treatment for laryngeal sarcoma. Radiation and chemotherapy are adjuvant therapies, usually kept as a salvage therapy after recurrence (9). In this case, the patient received treatment based on the existing protocols, for RRP and sarcoma (9).

Sarcoma cannot be directly related to the background of the patient. It is not possible to know whether there was a relationship between RRP/RRP treatment and malignant transformation or whether the sarcoma in this patient was coincidental.

## Conclusion

The heterogeneity of sarcomas with regard to molecular genetics, histology, clinical characteristics, and response to treatment makes management of this rare and varied neoplasm particularly challenging. Sarcoma of the larynx in a patient with recurrent respiratory papillomatosis is an extremely rare entity and this is the first report in the literature. 

## References

[1] Gillison ML, Alemany L, Snijders PJ, Chaturvedi A, Steinberg BM, Schwartz S, Castellsagué X ( 2012). Human papillomavirus and diseases of the upper airway: head and neck cancer and respiratory papillomatosis. Vaccine.

[2] Kanazawa T, Fukushima N, Imayoshi S (2013). Rare case of malignant transformation of recurrent respiratory papillomatosis associated with human papillomavirus type 6 infection and p53 overexpression. Springerplus..

[3] Pakkanen PP, Aaltonen LM, Sorsa TA, Tervahartiala Taina I, Hagström Jaana K, Ilmarinen Taru T (2019). Serum matrix metalloproteinase 8 and tissue inhibitor of metalloproteinase 1: Potential markers for malignant transformation of recurrent respiratory papillomatosis and for prognosis of laryngeal cancer. Head Neck..

[4] Hamza AH, Nasr MM, Deghady AA (2005). The use of mitomycin-C for respiratory papillomas: clinical, histologic and biochemical correlation. Saudi Med J..

[5] Egbert J D, Frederik G (2010). Topical use of MMC in the upper aerodigestive tract: a review on the side effects. Eur Arch Otorhinolaryngol.

[6] Agrawal N, Morrison GA (2006). Laryngeal cancer after topical mitomycin C application. J Laryngol Otol..

[7] Ramdulari AV, Izzuddeen Y, Benson R, Mallick S, Venkatesulu B, Giridhar P (2021). Laryngeal soft tissue sarcoma: A systematic review and individual patient data analysis of 300 cases. Head Neck.

[8] Mantilla JG, Xu H, Ricciotti RW (2020). Primary Sarcomas of the Larynx: A Single Institutional Experience with Ten Cases. Head Neck Pathol..

[9] Liu W, Jiang Q, Zhou Y (2018). Advances of systemic treatment for adult soft-tissue sarcoma. Chin ClinOncol..

